# Microwave-Assisted Synthesis of an Alternant Poly(fluorene–oxadiazole). Synthesis, Properties, and White Light-Emitting Devices

**DOI:** 10.3390/polym11101562

**Published:** 2019-09-25

**Authors:** Dumitru Popovici, Andrei Diaconu, Aurelian Rotaru, Luminita Marin

**Affiliations:** 1 ”Petru Poni” Institute of Macromolecular Chemistry, Romanian Academy, 700487 Iasi, Romania; dumitru.popovici@icmpp.ro; 2 Faculty of Electrical Engineering and Computer Science, Stefan cel Mare University, 720299 Suceava, Romania; andrei.diaconu@usm.ro (A.D.); aurelian.rotaru@usm.ro (A.R.)

**Keywords:** microwave, conjugated polymers, white organic light emitting diode, H-aggregates

## Abstract

An alternant poly(dihexyl fluorene-co diphenyl oxadiazole) has been synthetized by microwave-assisted oxidative polymerization. The structure has been confirmed by ^1^H-NMR and FTIR spectroscopies. Gel permeation chromatography indicated high molecular weight and low polydispersity index. DFT calculations suggested a complete separation of HOMO and LUMO orbitals, which were located on fluorene and oxadiazole moiety, respectively. X-ray diffraction, polarized light microscopy, and atomic force microscopy indicated the polymer tendency to stack into a layered morphology with a more compact structure for the films prepared by spin coating. Furthermore, UV-vis and photoluminescence spectroscopies indicated the formation of H-aggregates which played a key role in photoluminescence quenching in solid state. Nevertheless, the good charge mobility gained due to the orbital overlapping in H-aggregates led to excellent electroluminescence, which enabled the development of white OLED devices with outstanding stability.

## 1. Introduction

Light emitting diodes based on organic materials (OLEDs) stimulated extensive research worldwide in an effort to attain efficient organic electroluminescent materials with high lifetime and efficiency. One approach in reaching this goal is designing π-conjugated polymers, known to have distinct over inorganic materials. They are cheaper, lighter, and can be processed from solution or molten state, either as thin films or complex patterns, by simple casting, inkjet printing, or lithographic processes, opening new opportunities towards flexible devices. They can be operated with low DC voltages, have uniform luminosity, and, maybe the most important benefit, they allow the tuning of the emission color by structural design [1,2,3,4,5].

Among the conjugated polymers, the electroactive and photoactive polyfluorene is recognized as a reference polymer in OLED development, leading to devices with high efficiency and stability. It is a versatile blue light emitting polymer, whose emission color can be easily tuned by structural engineering or even simple doping, offering multiple possibilities for the preparation of materials with tuned properties [6,7,8,9,10]. Attempts to improve the polyfluorene properties by molecular engineering revealed that connecting the fluorene unit with higher electron affinity moieties enabled a balanced charge injection and transport, which significantly augmented the electroluminescence properties [11,12]. In this line of thought, great findings were achieved by joining the electron donating fluorene with building blocks containing the electron withdrawing oxadiazole [13]. An overview of the literature data revealed that oxadiazole and fluorene-based chromophores were joined by a randomly [14,15,16] or alternately [17,18,19,20,21] manner by Suzuki or Still polycondensation, in various mixtures with other aromatic/heterocyclic moieties. Besides, polyfluorene with oxadiazole pendant units [22,23,24] or low molecular weight compounds [25,26,27] were also synthetized. The structure–performance relationship of these materials demonstrated that the oxadiazole chromophore plays a key role in improving the electroluminescence properties of the polyfluorene, leading to deeper blue light emission, or even white light.

The main approach to preparing π-conjugated polymers is Yamomoto or Suzuki direct coupling polycondensation in a multi-step laborious procedure including the manipulation of sensible functional groups, and the necessity of prohibitive cost metal catalysts, which are difficult to handle and make the purification a difficult task increasing more the production price. New synthetic procedures developed over the last years revealed the microwave-assisted synthesis as an appropriate method to overcome the disadvantages of the above-mentioned synthetic pathways [28,29,30]. Its use in the preparation of π-conjugated polymers yielded polymers of high molecular weight and low polydispersity. Moreover, it was proved that it is a suitable method for the preparation of π-conjugated polymers by oxidative polymerization even from monomers with low electron density, since the alternating electric field favors the local charge reorganization [31].

In this paper we report on the synthesis of the poly(dihexyl fluorene-co-diphenyl oxadiazole) by microwave-assisted oxidative polymerization, its properties, and building of an organic light emitting diode. Poly(dihexylfluorene) has been obtained in similar conditions in order to properly asses the structural characterization data and the photophysical properties.

## 2. Experimental

### 2.1. Materials

Benzoic acid (99.5%), hydrazine monohydrate (98%), fluorene (98%), 1-bromohexane (98%), tetrabutyl-ammonium (97%), sulfuric acid (99.9%), phosphorous oxychloride (99%), and sodium hydroxide (97%) were purchased from Sigma-Aldrich (Saint Louis, MO, USA) and used as received. Ethanol (98%) was purchased from Carl Roth (Karlsruhe, Germany) and dried on molecular sieves before use.

### 2.2. Synthesis

#### 2.2.1. 2,5-diphenyl-1,3,4-oxadiazole (**DPO**)

**Scheme 1:**
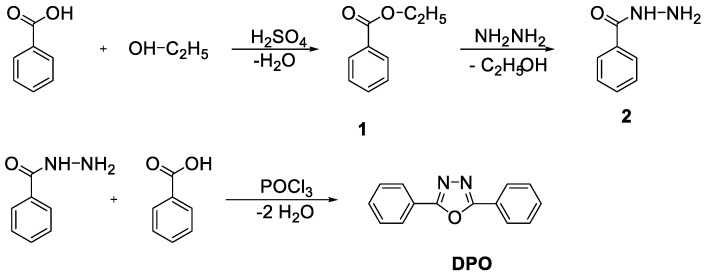
Synthesis of 2,5-diphenyl-1,3,4-oxadiazole (DPO) monomer.

The synthesis of 2,5-diphenyl-1,3,4-oxadiazole was performed according to a protocol reported in literature, as follows [32,33]. In a round bottom flask, 20 g (0.164 mol) benzoic acid, 100 mL dry ethanol, and 3 mL H_2_SO_4_ were introduced. The obtained solution was vigorously stirred under gentle reflux for 12 h, and then concentrated by rotary evaporation. The crude product was dissolved in diethyl-ether and the sulfuric acid was neutralized with a 5% sodium carbonate solution until no bubbles were observed. The organic phase was extracted then in a separation funnel, washed with water, dried on magnesium sulfate, and concentrated by rotary evaporation to give 19.6 g (η = 79.5%) of yellow liquid, identified by NMR as the ester **1**. Further, the ester **1** (19.54 g, 0.13 mol) was mixed with 6.5 mL (0.13 mol) hydrazine monohydrate and heated at gentle reflux in ethanol for 36 h to obtain 8.94 g (65.6 mmol, η = 50.5%) of hydrazide **2**, as yellowish needle crystals. The hydrazide **2** was then reacted with benzoic acid, in stoichiometric amounts, as follows: 8.94 g (65.6 mmol) of hydrazide **2**, 8.01g (65.6 mmol) of benzoic acid, and 7 mL POCl_3_ were introduced into a round bottom flask and heated at reflux for 16 h. The reaction mixture was poured into a crushed ice vessel and neutralized with a sodium carbonate solution (5%), when the product precipitated as an ochre powder. It was filtered off, washed with hot water, and dried under vacuum for 24 h. The crude product was recrystallized from a mixture of DMF/Ethanol, 1/1. η = 25.5% (3.74 g), p.t. 138 °C. The reaction pathway was given in Scheme 1.

^1^H-NMR (CDCl_3_, 400 MHz): δ = 8.2, 8.1 ppm (d, 4H, Ar-H), 7.6–7.5 ppm (superposed signals, 6H, Ar-H). FTIR (KBr): 3060–2920 cm^−1^ (νC-H aromatic), 1991–1767 (δC-H aromatic) 1606 cm^−1^ (νC=N), 1213 cm^−1^ (νC-O-C), 1586, 1548 (νC=C of aromatic ring), 709, 687 (ν monosubstituted aromatic ring).

#### 2.2.2. 9,9-dihexyl-fluorene (**DHF**)

**Scheme 2:**
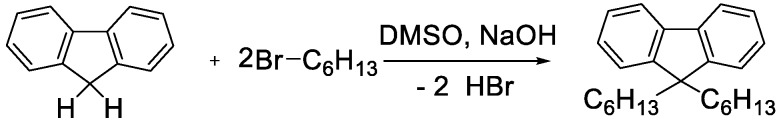
Synthesis of 9,9-dihexyl-fluorene (DHF) monomer.

In a round bottom flask 40 mL DMSO, 3.32 g (20 mmol) fluorene, and 0.332 g (1 mmol) tetrabutyl-ammonium were sequentially introduced under nitrogen inert atmosphere and vigorous stirring. The solution was heated to 80 °C, and then 12 mL NaOH 50% and 7.2 mL (52 mmol) of 1-bromohexane were added successively by slowly dropping. The system was kept at 80 °C under vigorous stirring for 16 h. The reaction mixture was then poured into water, extracted in a separation funnel with dichloromethane, washed with saturated brine, dried on magnesium sulfate, and concentrated by rotary evaporation. The crude product was purified by column chromatography by eluting with heptane. The product was obtained as a transparent yellowish liquid. η = 95.7% (4.48 g) (Scheme 2).

^1^H-NMR (CDCl_3_, 400 MHz): δ = 7.7 (d, 2H, Ar-H), 7.2–7.3 (superposed signals, 6H, Ar-H), 1.9–2.0 (t, 4H, -CH_2_-) 1.0–1.1 (superposed signals, 12H, -CH_2_-), 0.7-0.8 (t, 6H, CH_3_), 0.6 (m, 4H, CH_2_). FTIR (KBr): 2955–2855 cm^−1^ (νC-H aliphatic), 3066–3015 cm^−1^ (νC-H aromatic), 1604 cm^−1^ (νC = C of aromatic ring).

#### 2.2.3. MW-assisted Synthesis of Poly(dihexyl fluorene-co diphenyl oxadiazole) (**POF**)

**Scheme 3:**
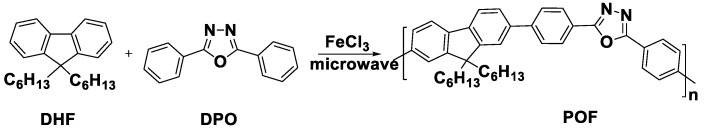
Synthesis of poly(dihexyl fluorene-co diphenyl oxadiazole) (**POF**).

The polymer was synthesized using the microwave technique, by reacting 2,5-diphenyl-1,3,4-oxadiazole and 9,9-dihexyl-fluorene monomers in the presence of FeCl_3_ catalyst (Scheme 3) [3]. In order to optimize the reaction conditions, a series of tests were performed regarding on a mixture of 1/1 fluorene/oxadiazole monomers, by varying the irradiation power, reaction time, and working temperature. For each test, the molecular weight was measured and the polymerization yield was calculated. The reaction conditions for the best molecular weight/yield balance were: irradiation time: 25 min, power: 20 W, temperature: 50 °C. A typical reaction procedure was as follows.

In a standard Pyrex vessel sealed with a Teflon septum cap, 0.1 mmol of 9,9-dihexyl-fluorene (33.4 mg), 0.1 mmol 2,5-diphenyl-1,3,4-oxadiazole (22.2 mg), 0.13 g of FeCl_3_, and 4 mL CHCl_3_ were introduced, under a nitrogen atmosphere. The vessel was introduced into the microwave equipment and the established conditions (irradiation time: 25 min, power: 20 W, temperature: 50 °C) were applied. Then, the reaction mixture was precipitated in 50 mL solution of CH_3_OH: HCl (10:1, v/v), filtered of, dissolved in chloroform, washed thrice with HCl solution 5%, washed thrice with water, filtered on Celite, and then concentrated by rotary evaporation. In order to narrow the polydispersity index, the crude product was further extracted with n-heptane for 12 h in a Soxhlet apparatus, and then washed with methanol. This reaction protocol has been repeated 10 times, in order to obtain a reasonable amount of sample for further experiments. Each time, an amount of polymer comprised between 28 and 33 mg was obtained. The reaction yields and molecular weight were given in Table 1.

With the aim to prepare a series of polymers with different content of oxadiazole, the same reaction conditions were applied for three different ratios of monomers, **DHF**/**DPO** = 1/1; 2/1; 3/1, and the obtained polymers were coded **POF1**, **POF2**, **POF3** (Table 1). The reaction yield, the value of molecular weight, and ^1^H-NMR spectra indicated that in each case the reaction proceeded for a 1/1 molar ratio of the two monomers, implying that this kind of polymerization led to an alternant polymer. The reaction yield and molecular weight for these different molar ratios were given in Table 1.

Polyfluorene was prepared by a similar procedure in order to have a reference polymer.

### 2.3. Equipment

FTIR spectra were recorded on a Bruker Vertex 70 spectrophotometer (Ettlingen, Germany), in the transmission mode, by using KBr pellets.

^1^H-NMR spectra were acquired on a Bruker Avance DRX 400 MHz Spectrometer (Rheinstetten, Germany) equipped with a 5 mm QNP direct detection probe and z-gradients. The samples were prepared in deuterated chloroform and the spectra were registered at room temperature. The chemical shifts were reported as δ values (ppm) relative to the residual peak of the solvent.

Solubility tests were performed by dissolving 10 mg sample into 1 mL solvent.

Molecular weight distributions of the polymers were measured by gel permeation chromatography (GPC) method on a PL-EMD 950 Evaporative Light Scattering Detector instrument, using chloroform as eluent and standard polystyrene samples (60,000) for calibration. The measurements were carried out at room temperature.

The thermotropic behavior of polymers was investigated with an Olympus BH-2 polarized light microscope (Center Valley, PA, USA) under cross polarizers equipped with a THMS 600 hot stage and a LINKAM TP92 temperature control system.

UV–Vis absorption and photoluminescence spectra were recorded on a Carl Zeiss Jena SPECORD M42 spectrophotometer (Jena, Germany) and a Perkin Elmer LS 55 spectrophotometer (Beaconsfield, UK), respectively, in solution (10^−7^ M), using 10 mm quartz cells. The quantum yield (Φ_F_) of the samples, in solution and films, was registered on a FluoroMax-4 spectrofluorometer equipped with a Quanta-phi integrating sphere Horiba Jobin Yvon, at room temperature. The solution concentration was optimized to obtain an absorbance around 0.055. The slit widths and detector parameters were optimized to maximize but not saturate the excitation Rayleigh peak, in order to obtain a good optical luminescence signal-to-noise ratio.

Atomic force microscopy (AFM) topography and phase contrast images have been recorded using a NT-MDT Solver Pro-M scanning probe microscope (Renishaw, UK) in non-contact mode.

Polarized light microscopy of the POF as powder and filmw, performed with an Olympus BH-2 polarized light microscope (Center Valley, PA, USA) equipped with with a THMS 600 heating stage and a LINKAM TP92 temperature control system.

A WS-650 Laurell Spin Coater was used for preparation of the thin films.

#### Organic Light Emitting Device Preparation

To establish the optimal conditions for building organic light emitting diodes, devices were built in three different phases, applying various conditions, which were described in the supporting information. The conditions under which the OLED devices with the best performance were obtained are described as follows.

The OLEDs were built into a glove box on pre-patterned indium-tin oxide (ITO) support with a sheet resistivity of 8–12 Ω/m^2^. Before use, the ITO supports were cleaned by successive ultrasonication in acetone, isopropanol, and methanol, and then covered with a thin layer of toluene which was removed by spin coating (12,000 rot/min). All the solvents were filtered with a PTFE 0.2 µm filter before use to avoid any contamination. All the solutions used for OLED building were ultrasonicated for 12 h at 30 °C and filtered with a PTFE 0.2 µm filter before use. On the ITO support, a primary layer of PEDOT-PSS 1% in water was deposited by spin coating at 2000 or 3000 rot/min at an acceleration of 4000 rpm/s/s, and stored in a vacuum oven at 100 °C before use. The organic layer was later deposited by spin coating a 1% solution of **POF** in chlorobenzene, using various rotation speeds. Further, an aluminum cathode layer was deposited by thermal evaporation method through a mask to reach cross-bar electrode geometry. The aluminum film thickness was 100 nm. The devices were tested by using gallium–indium eutectic as an liquid drop electrode on both the area covered by the aluminums cathode, as well as directly on the area of **POF** polymer only.

Current–luminance–voltage (I–L–V) characteristics were measured with Keithley 2400 Source Meter (max. 20 V) equipment and a Si photodiode, which was placed immediately under the ITO glass support. Luminance was calibrated considering that 1 Cd generates 0.5 V. Electroluminescence spectra were recorded by an InstaSpec IV CCD spectrograph.

## 3. Results and Discussions

### 3.1. Structural Characterization

**Figure 1:**
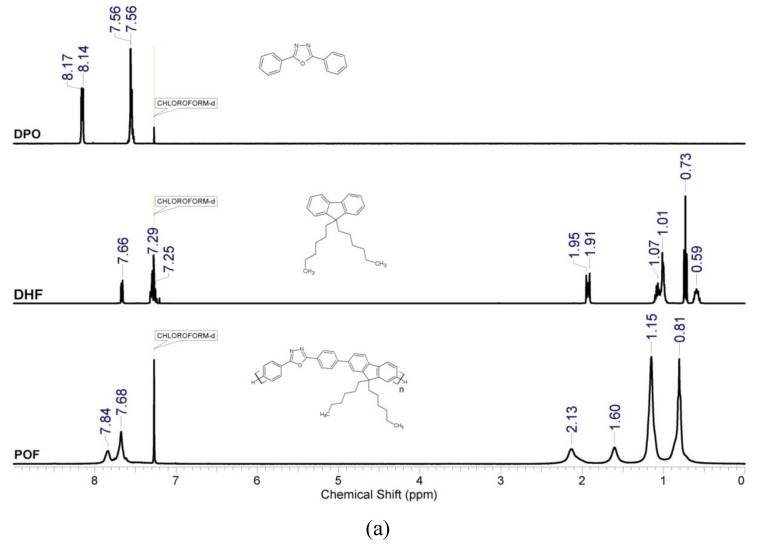

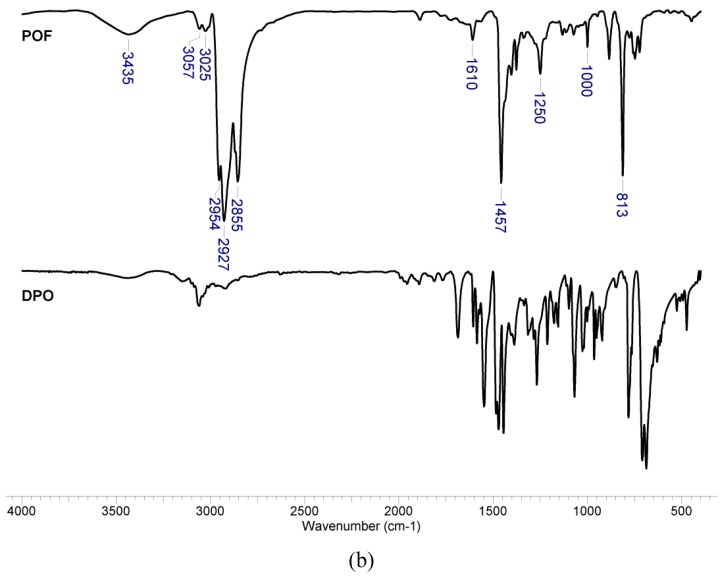
^1^H-NMR (**a**) and FTIR (**b**) spectra of the **POF** polymer and precursor monomers.

An alternant conjugated polymer based on fluorene and oxadiazole chromophores was synthetized by microwave technique. The structure of the new poly(fluorene–oxadiazole) has been confirmed by ^1^H-NMR and FTIR spectroscopy, which also indicated a high conjugation degree. A comparative analysis of the ^1^H-NMR spectra of the monomers and polymer revealed important structural aspects (Figure 1a). First, the strong electron withdrawing oxadiazole unit of **DPO** strongly influenced the chemical shifting of the neighbor aromatic protons which gave a doublet at 8.2 ppm, while the other protons gave superposed bands around 7.6 ppm. The conjugated fluorene unit of **DHF** gave the chemical shifting of its aromatic protons as a doublet at 7.7 and superposed bands at 7.3 ppm. No differences into ^1^H-NMR spectra were observed when the molar ratio between **DPO** and **DHF** was varied, (**POF1**, **POF2**, and **POF3**), and further the code **POF** will be used. The chemical shifting of the **POF** polymer protons appeared as two broad superposed bands with maxima at 7.8 and 7.7 ppm, at lower magnetic field compared to the aromatic protons neighboring oxadiazole and higher magnetic field compared to the aromatic protons of fluorene. Moreover, they were slightly shifted to higher magnetic field even when compared to the reference polyfluorene, which can be related to a better conjugation. All these data indicate on one hand the presence of fluorene and oxadiazole building blocks into the polymer chains, and on the other hand similar electronic vicinity of the protons, even if they belong to oxadiazole or fluorene units, suggesting a high conjugation of the two chromophore units. This should occur when the oxadiazole and fluorene units are placed in an alternant manner, as the data from their synthesis suggested (Table 1). Second, a similar observation was valuable for the FTIR spectra. The complex aspect of the finger print region of both oxadiazole and fluorene monomers caused by the vibration bands of the aromatic C=C bonds of aromatic rings was replaced by an intense band at 1457 cm^−1^, once again confirming the energy equalization of the bonds given by the strong conjugation of the two monomers. Moreover, the vibration band of the C=N bond appeared as a lower intensity band at 1610 cm^−1^, in accordance with its les preponderance compared to the increased number of C=C bonds from both monomers. The FTIR spectra also revealed the characteristic vibration of the p-substituted benzene ring at 813 cm^−1^ [34], confirming the presence of the aromatic ring of **DPO** into the polymer backbone, while the intense vibration bands of aliphatic CH_2_ and CH_3_ units from **DHF** appeared around 2927 cm^−1^. All these data confirmed the obtaining of the alternant poly(fluorene–oxadiazole) polymer.

The polymer rapidly solubilized in chloroform and chlorobenzene, and slowly dissolved in toluene, dimethylformamide, dimethylsulfoxide, N-methylpirolidone dichlormetahane, and tetrahydrofurane. No solubilisation was observed in acetonitrile and methanol.

### 3.2. Polymer Morphology

**Figure 2:**
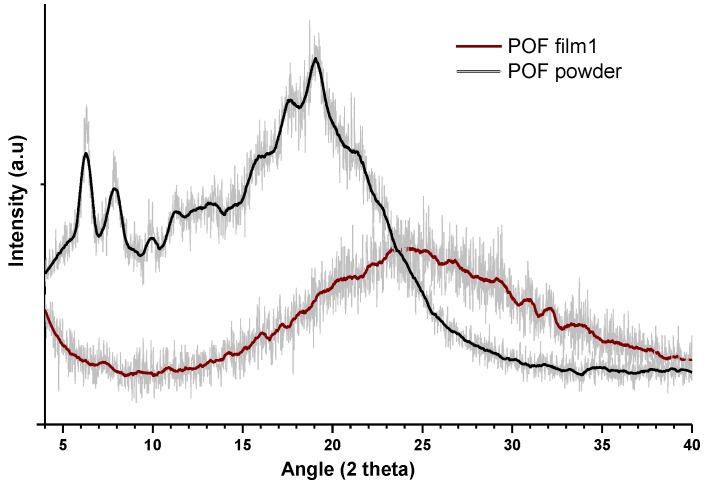
X-ray diffraction of the **POF** as powder and film.

X-ray diffractogram recorded on the **POF** powder showed the pattern characteristic of a semicrystalline polymer, mainly consisting of sharper reflections on the top of a broad halo (Figure 2) [35]. Two sharper reflections at 6.3 and 7.8° indicated a layered morphology, which is characteristic for the polymers with rigid backbones and lateral flexible chains [36,37]. The corresponding d-spacing of 14.7 and 11.3 Å, respectively, are in concordance with the inter-layer distances induced by the different arrangement of the alkyl chains between the rigid layers. The wider-angle domain was dominated by a broad halo from 10 to 30°, with many reflections around a more intense one at 19.04° corresponding to a d-spacing of 4.6 Å. It can be assumed that this pattern was prompted by a polydispersity of inter-molecular distances, from 8.8 to 3 Å, with a predominant value of 4.6 Å.

The diffraction profile drastically changed for the thin films casted from chloroform solution. The sharper reflections at lower angle were still present and slightly shifted to 6.2 and 7.3°, corresponding to a d-spacing of 14.2 and 12.1 Å, respectively. Compared to the powder diffractogram, the reflection corresponding to the inter-layer distance of 12.1 Å became predominant, indicating a more compact morphology of the films. The sharper reflections above the broad halloo almost disappeared and the reflection maximum significantly shifted to wider angle, at 24.3°. This also converges on the hypothesis of a more compact morphology, in which the predominant inter-molecular distance among the rigid chains is 3.7 Å. This means that the electron clouds of neighbour backbones are close enough to allow the electron jump between them, enabling the transverse conduction [38,39].

**Figure 3:**
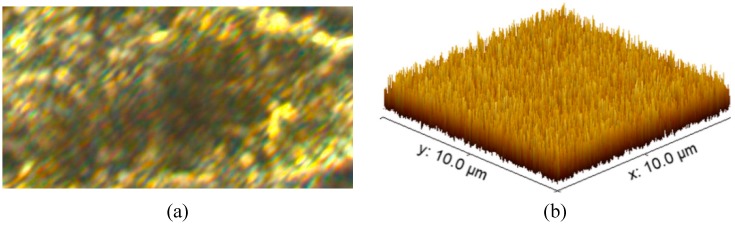

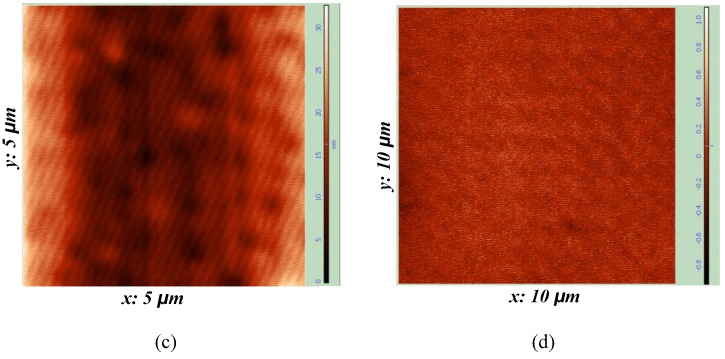
Representative polarized light microscopy (POM) (**a**), and atomic force microscopy (AFM) topography (**b**,**c**) and phase contrast (**d**) images of the **POF** film casted from chloroform solution.

A complementary proof of the supramolecular ordering was provided by polarized light microscopy (POM) on the polymer powder and films, which revealed a birefringent banded texture, characteristic to the lamellar morphology of the side-chain polymers (Figure 3a) [37,40]. Heated at a rate of 10 °C/min, the polymer melted at 117 °C when a viscous isotropic state was reached. The further cooling evidenced the polymer freezing in amorphous state, in concordance with the rigidity of its backbone, which hindered the chain mobility thus preventing the crystallization [41,42,43].

To further analyse the film quality, topographic and phase contrast atomic force microscopy (AFM) images of the **POF** films were acquired (Figure 3b–d). As it can be seen, the films are quite rough at the nanometric level, the root mean square roughness being around 121 nm over a 10 µm^2^ scan area, in agreement with the semicrystalline nature of the polymer (Figure 3b). Interestingly, the banded texture observed in POM was present at the nanometric level as well, confirming once more the layered supramolecular ordering of the **POF** (Figure 3c). Contrast phase images revealed small values of the phase contrast shift, indicating relief differences but not chemical composition variation (Figure 3d).

### 3.3. Photophysical Behavior

The targeted polymer has been designed in order to yield luminescent appropriate materials for use as emissive semiconductor layer of OLED devices. To this end, the photophysical behaviour of the **POF** polymer has been investigated by UV-vis and photoluminescence spectroscopies.

**Figure 4:**
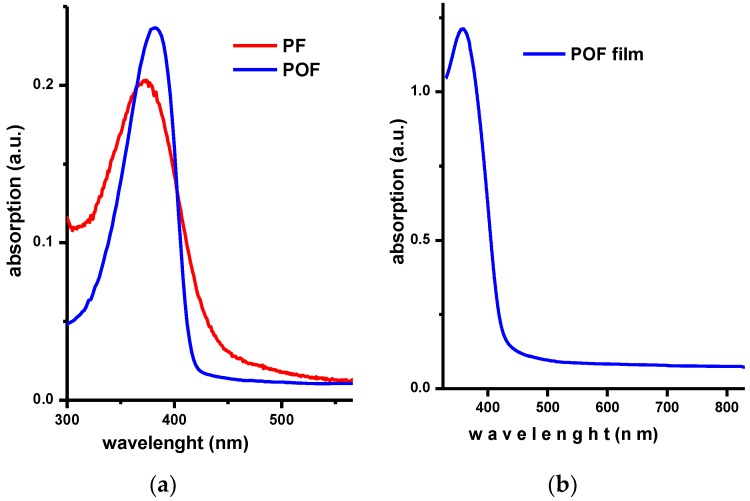
UV-vis spectra of the polyfluorene (**PF**) and poly(fluorene–oxadiazole)—**POF**, in solution (**a**) and film (**b**).

The electronic properties of the **POF** polymer were investigated by UV-vis spectroscopy, in a diluted chloroform solution of 10^−7^ M, when the macromolecules can be considered isolated and unaffected by aggregation (Figure 4a). The polymer gave a sharp absorption band between 300 and 400 nm, with an absorption maximum at 384 nm, attributed to the π–π* transition of the localized conjugated system. Compared to the reference polyfluorene, the absorption maximum of **POF** was bathochromic shifted 10 nm, reflecting the better conjugation enabled by the fluorene–oxadiazole donor–acceptor system. Looking at the absorption band, it can be remarked that the poly(fluorene–oxadiazole) has a sharper profile compared to that of the polyfluorene, suggesting less conformational isomers in solution in concordance with a better conjugation and consequently with a higher stiffness of the polymeric chains [44]. Applying the energy equation of quantum mechanics to the wavelength corresponding to the absorption maximum (384 nm), the value of the optical bandgap of the **POF** molecules was calculated to be 3.22 eV compared to 3.31 eV found for **PF** [45]. The decreasing of the HOMO–LUMO energy by conjugation of the directly connected electron withdrawing oxadiazole with the electron donating fluorene was obvious. In solid state, the absorption profile of **POF** preserved its sharp shape, but the absorption maximum was drastic hypsochromic shifted to 359 nm (Figure 4b). This phenomenon is common for the n-type conjugated polymers with planar configuration, which have the tendency to form H-aggregates with dense packing and π orbital overlap [46], as the X-ray diffraction indicated.

**Figure 5:**
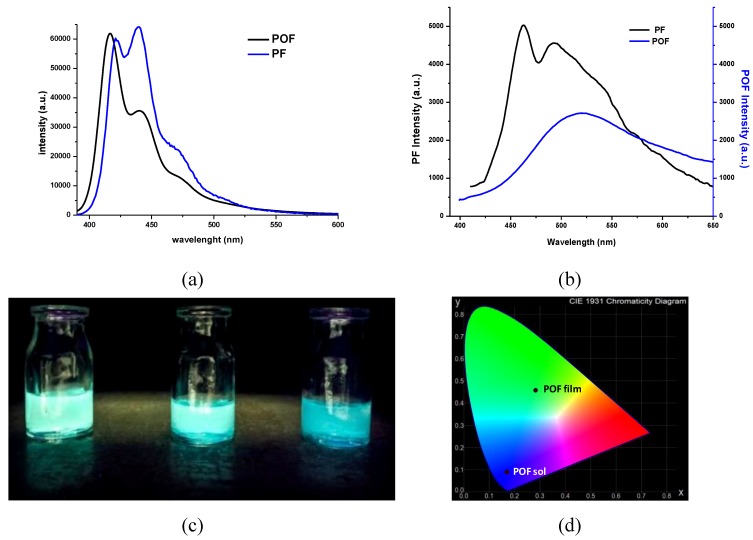
Emission spectra of the poly(fluorene–oxadiazole) and polyfluorene (**a**) in solution and (**b**) film; (**c**) Images of POF solution of 10^−5^, 10^−6^, 10^−7^ M (from left to right) under an UV lamp; (**d**) chromaticity diagram.

The emissive properties of the **POF** were prior assessed by photoluminescence spectroscopy, in solution and solid state, by comparison with the reference **PF** polyfluorene. By exciting with light of energy corresponding to the absorption maxima (3.22 eV and 3.31 eV, respectively) both solutions gave a quite similar emission profile, in the blue spectral domain from 400 to 490 nm, consisting in the superposition of three emission bands, with the maxima around 417 nm, 440 nm, and 473 nm, respectively (Figure 5a). The spectrum of the **POF** was slightly blue shifted compared to that of **PF**, in agreement with the presence of the oxadiazole blue light emitter. Different emission behaviour was noticed in solid state. The reference polyfluorene maintained the emission profile registered in solution, consisting of three superposed emission bands, but covering a larger spectral domain (from 400 to 650 nm), with the two principal maxima shifted to 451 and 482 nm. On the contrary, the solid-state poly(fluorene–oxadiazole) displayed a single large emission band extended throughout the entire visible domain, from 450 to 700 nm, with an emission maximum in the green spectral domain, at 512 nm. This drastic red shifting of the emission maximum in thin film versus solution was attributed to the higher coplanarization degree of the chains in solid state, when the rotational intramolecular moving is restricted [47]. Moreover it is known that the supramolecular ordering favours the formation of excimers, which emit at a larger wavelength leading to a band tailing [48]. This hypothesis was supported by X-ray diffraction measurements, which indicated dense macromolecules packing in solid state. Moreover, the images of **POF** solutions, when excited under an UV lamp, showed shifting of the colour of the emitted light from blue to whitish green when the concentration varied from 10^−7^ to 10^−5^ M, indicating the aggregation influence (Figure 5c). The colour change of emitted light was also observed in the chromaticity diagram, which displayed shifting of the blue light emission in solution to whitish green in solid state (Figure 5d).

This emissive behaviour was reflected in the value of the quantum yield as well. As expected, the microwave synthetized polyfluorene showed a high quantum yield in solution, of 68%, when excited with the maximum absorption of 374 nm, and the new poly(fluorene–oxadiazole) also showed a high quantum yield in solution, of 64%, when excited with the maximum of absorption of 384 nm. In solid-state, the **PF** showed a high quantum yield of 19%, but the quantum yield of **POF** drastically diminished to 8.5%. This was in agreement with the more planar structure of the **POF** backbones, which facilitated a layered ordering in solid state, favourable for non-radiative deexcitation by excimer formation [49]. A decrease of the quantum efficiency induced by the presence of oxadiazole has been also observed in the reported literature and was ascribed to the nitrogen and oxygen atoms considered as quenching sites of the copolymers [17].

### 3.4. Theoretical Calculations

**Figure 6:**
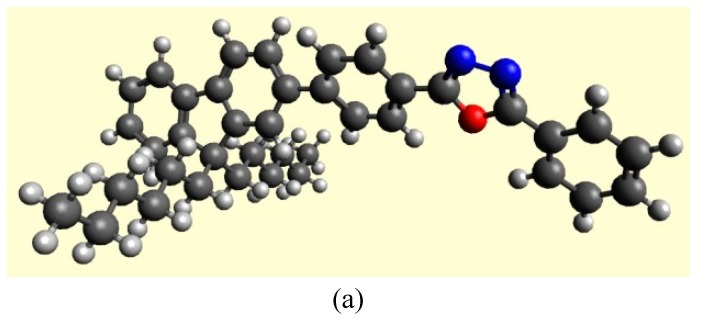

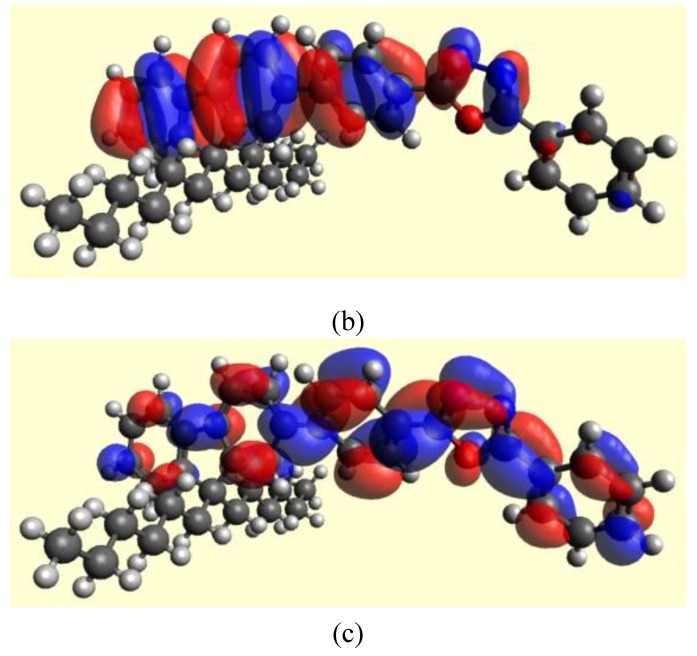
Optimized geometry of a structural unit (**a**), and the spatial distribution of the HOMO (**b**), and LUMO orbitals (**c**).

**Figure 7:**
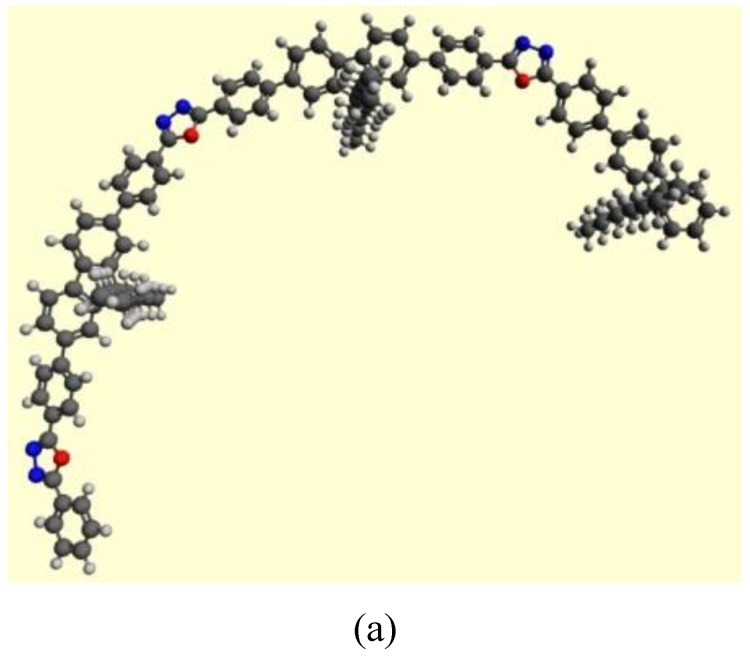

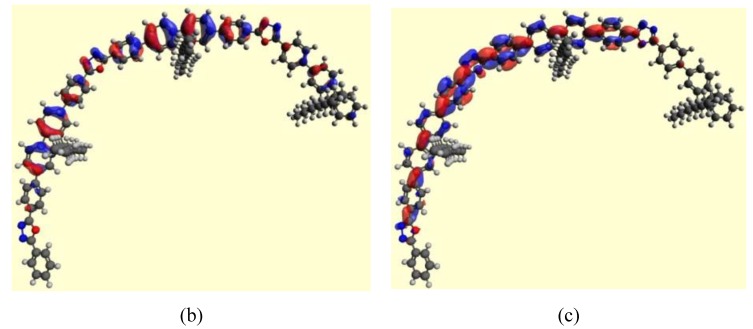
Optimized geometry and spatial distribution of chain segment consisting of three structural units (**a**), and spatial distribution of HOMO (**b**) and LUMO (**c**) orbitals.

To correlate the polymer design with its photophysical properties, geometrical, and electronic properties for both (a) one structural unit of polymer (Figure 6) and (b) one chain segment made up of three structural units (Figure 7), were theoretically calculated. The calculations were performed using the density functional theory DFT B3LYP method at 6-31G (d,p) level of theory implemented in GAMESS-US software [50,51]. Minimum energy conformations and HOMO and LUMO molecular orbitals on the structural unit are presented in Figure 6. For the structural unit, an almost complete separation of the HOMO and LUMO levels was noted, HOMO orbitals being mainly located on the fluorene moiety (E_HOMO_= −6.0927eV), while LUMO orbitals were located in particular on the oxadiazole segment (E_LUMO_= −2.0599eV) (Figure 6). Similar data were also reported for compounds containing these two building blocks [52].

In the chain segment consisting from three structural units, the separation of the HOMO and LUMO levels was present as well. Compared to that of the structural unit, it appeared to be less marked, possible due to an intramolecular charge transfer (Figure 7). This distribution of the molecular orbitals suggests that they can efficiently assure the charge-carrier transport through both *n*-type and *p*-type conduction, and prevent the reverse energy transfer [53]. This is particular important for OLED device engineering, as it permits the elimination of an additional compensation layer of the load carriers.

### 3.5. OLED Devices

OLED devices were constructed based on **POF** as the active substrate. In order to optimize the conditions of OLED preparation, devices were built varying a series of parameters, such as: the solvent used for the preparation of the **POF** film; the annealing temperature; spinning rate; and cathode nature (Tables S1 and S2, Figure S1). The analysis of the current–luminance–voltage curves (Figures S2–S4) revealed that the best performances were obtained for the following conditions. (i) **POF** films were manufactured from solutions in a solvent with higher boiling point, at a slower spinning rate, when thicker continuous films, without cracks or holes, were obtained. (ii) Gallium–indium eutectic and aluminium proved to have the most appropriate work function for **POF** polymer. The aluminium electrode deposited by thermal evaporation showed the best results, most probably because this method succeeded to generate a continuous electrode film on the rough surface of the semicrystalline **POF** substrate. (iii) The polymer substrate was annealed at a temperature lower than the melting point. Considering the POM observation, it can be concluded that the electroluminescence was favoured by the better charge mobility of the semicrystalline morphology *versus* the amorphous state. (iv) The use of PEDOT:PSS did not substantially improved the luminance, but on the contrary, the electroluminescence intensity was almost one order of magnitude higher for the direct **POF**–**ITO** contact. This was quite an intriguing observation taking into consideration that PEDOT:PSS is usually required for OLED devices in order to minimize the hole injection barrier. This indicates that the cooperative effect of the good separation of the HOMO–LUMO orbitals and supramolecular layered ordering of the POF favoured an efficient charge transfer at the organic–inorganic (**POF**–**ITO**) junction.

**Figure 8:**
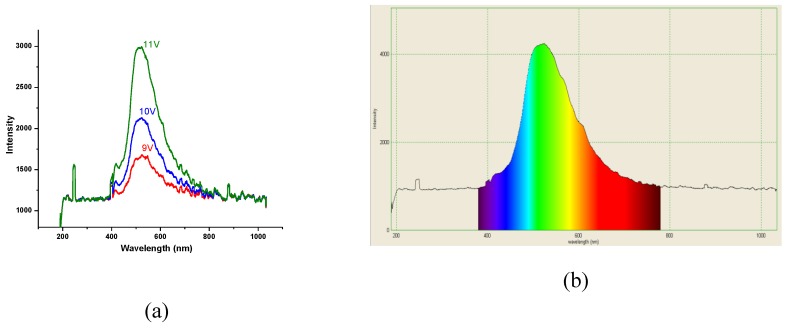
Electroluminescence spectra of light emitting diodes based on organic materials (OLED) devices (ITO/PEDOT:PSS/POF/Ga–In) (**a**) when excited with 9, 10, and 11 V (sample 1 in supporting information); (**b**) measured at 11 V (sample 5 in supporting information).

The functional OLEDs showed emission on the entire visible spectral domain, with a maximum in the green region (Figure 8). The shape of the electroluminescent spectrum was similar to the photoluminescent one, but the emission maximum was red shifted to 525 nm and emission profile was slightly broader. Compared to other cases reported in literature, in which a significant broadening of the electro-luminescence spectrum compared to the photo-luminescence one has been recorded [13,17,54,55], the preservation of an almost similar emission profile, either by photo-excitation or by electro-excitation, points to a good morphological stability of the active substrate under the influence of the electric field, an important aspect for the OLED applications. The electroluminescence spectrum recorded for different applied voltages, displayed an increase of luminescence intensity with applied voltage value, reaching an almost double intensity when the voltage increased from 9 to 11 V (Figure 8a).

**Figure 9:**
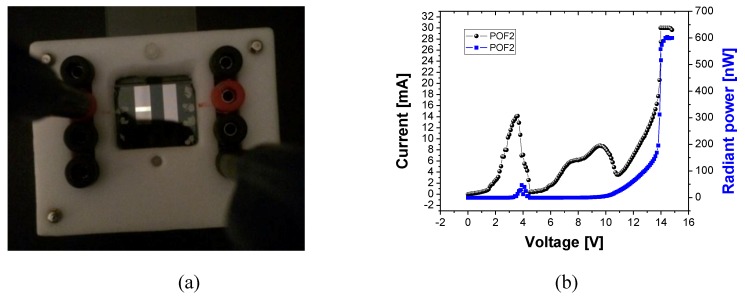
The image of the OLED device (**a**) and current–luminance–voltage curve for ITO/PEDOT:PSS/POF/Al device (**b**).

All OLED devices built in a clean room, with aluminium cathode, showed white light emission (Figure 9a). A current–luminance–voltage curve is given in Figure 9b. Interestingly enough, the devices presented a stable emission, without degradation of the colour of emitted light for more than one week, proving an excellent lifetime.

## 4. Conclusions

A poly(fluorene–oxadiazole) polymer has been prepared using the microwave-assisted synthesis for the first time. This method showed the advantages of easy synthesis in mild reaction conditions of polymers of high molecular weight. The as-prepared polymer has an alternant structure which favoured the ordering in a dense structure of H-aggregates, which quenched the photoluminescence and enhanced the electroluminescence. The combining of electron-withdrawing oxadiazole and electron-donation fluorene into an alternant structure led to an excellent electronic conjugation with a complete separation of the HOMO and LUMO levels. These characteristics of the polymer enabled an excellent electrode–polymer junction of the constructed OLED devices, which showed white light emission and extended lifetime.

## Figures and Tables

**Table 1:** Reaction yield and molecular weight of the polymers obtained for different molar ratios of the DFH/DPO monomers.

Code	DHF: DPO (mol/mol)	η (%)	Mn (g/mol)	Mw/Mn
PF	1:0	47	56 350	2.8
POF1	1:3	5.54	89 000	3.2
POF2	1:1	55.4	84 800	1.9
POF3	3:1	35.15	80 100	3.8

## Supplementary Materials

The following are available online at https://www.mdpi.com/2073-4360/11/10/1562/s1.
