# Supplementary material for: Microwave-Assisted Synthesis of an Alternant Poly(fluorene–oxadiazole). Synthesis, Properties, and White Light-Emitting Devices
Source: Polymers (Basel). 2019 Sep 25;11(10):1562. doi: 10.3390/polym11101562 (PMC6835352; doi:10.3390/polym11101562)
Supplement: Supplementary file 1 [file polymers-11-01562-s001.pdf]

Microwave-assisted synthesis of an alternant poly(fluorene-oxadiazole). Synthesis, properties and white light-emitting devices

Dumitru Popovici[1], Andrei Diaconu[2], Aurelian Rotaru[2], Luminita Marin[1*]

[1]"*Petru Poni*" Institute of Macromolecular Chemistry of Romanian Academy, Iasi, Romania

[2]"*Stefan cel Mare*" University, Fac. Elect. Eng. & Comp. Sci, Suceava, Romania

OLED building

To establish the optimal conditions for building organic light emitting diodes, devices were built in three different phases under various conditions, as follows.

Phase 1. The OLEDs were built on prepatterned indium-tin oxide (ITO) support with a sheet resistivity of 8-12 Ω/m^2. Before use, the ITO supports were cleaned by ultrasonication, firstly in chloroform and then in acetone. To improve the charge injection and minimize the leakage, an primary layer of 1% poly(ethylenedioxythiophene)-poly(styrenesulfonic acid) (PEDOT:PSS) in water was deposited on top of the ITO layer by spin coating (8000 rot/min at different accelerations) for 3-5 min, and subsequently thermally treated in an vacuum oven for 5 min at various temperatures (Table S1). The organic layer was then deposited by spin coating a 1% solution of **POF** in either chloroform or toluene, using various spinning rates and accelerations. The as obtain material was thermally annealed in a vacuum oven for 5 minutes at various temperatures and then subjected to the deposition of the electron injection cathode. Chromium or platinum were deposited by cathodic pulverization in an argon atmosphere through a mask. The resulting electrodes were 3 mm in diameter and 5 nm in thickness (Figure S1a). Aluminium was deposited by sputter coating method, as thin films of 80 nm thickness. For some of the devices, liquid gallium-indium eutectic was used as the superior electrode. 20 different devices were built. The conditions under which they were built were given in Table S1.

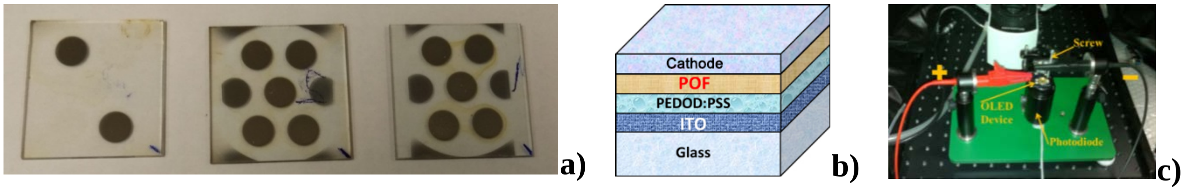

Figure S1. Images of a) electrodes devices; b) their schematic representation; and c) the experimental setup used for OLED testing

Table S1. Conditions used in building OLEDs in phase 1

**Sample**	**Solvent**	**PEDOT:PSS Speed/Acceleration /Annealing temperature**	**POF Polymer Speed/Acceleration /Annealing temperature**	**Superior electrode**	**Current-luminance-voltage curve**
**1**	Toluene	8000/100/100	8000/100/50	Ga-In	Fig.S1-a
**2**	Toluene	8000/200/100	8000/100/NA	Ga-In	Fig.S1-b
**3**	Toluene	8000/100/NA*	8000/100/NA	Ga-In	Fig.S1-c
**4**	Toluene	8000/100/NA	6000/30/NA	Ga-In	Fig.S1-d
**5**	Toluene	8000/100/NA	6000/50/NA	Ga/In	Fig.S1-e
**6**	Toluene	8000/150/80	8000/100/NA	Pt	Fig.S1-f
**7**	Chloroform	8000/150/80	6000/50/NA	Ga-In	
**8**	Chloroform	8000/150/80	6000/50/120	Ga-In	
**9**	Chloroform	8000/150/80	6000/50/NA	Cr	
**10**	Chloroform	8000/150/80	6000/100/NA	Cr	
**11**	Chloroform	8000/150/80	6000/200/NA	Cr	
**12**	Chloroform	8000/150/80	6000/200/80	Cr	
**13**	Toluene	8000/150/80	6000/50/NA	Pt	
**14**	Toluene	8000/150/80	6000/100/NA	Pt	
**15**	Chloroform	8000/150/80	3000/50/NA	Al	
**16**	Chloroform	6000/150/80	6000/50/NA	Al	
**17**	Chloroform	6000/150/80	4000/50/NA	Al	

**18**	Toluene	6000/150/80	4000/50/NA	Al	
**19**	Toluene	6000/150/80	5000/50/NA	Al	
**20**	Toluene	6000/150/80	4000/50/NA	Al	

*NA – not applied

Phase 2. The OLEDs were built on prepatterned indium-tin oxide support with a sheet resistivity of 8-12 Ω/m^2. Before use, the ITO supports were cleaned by successive ultrasonication in acetone, isopropanol and methanol, and then covered with a thin layer of toluene which was removed by spin coating (12000 rot/min) before use. All the solvents were filtered with a PTFE 0.2 µm filter before use to avoid any contamination. All the solutions used for OLED building were ultrasonicated for 12 hours at 30 ^oC and filtered through a PTFE 0.2 µm filter before use. Over the ITO surface a primary layer of PEDOT-PSS 1% in water was deposited by spin coating at either 2000 or 3000 rot/min and an acceleration of 4000 rpm/s/s, and stored in a vacuum oven at 100 ^oC before use. The organic layer was then deposited then by spin coating of a 1% solution of **POF** in chlorobenzene, using various rotation speeds and annealing temperatures (Table 2s). Further, the aluminium cathode was deposited by sputter coating method as thin films of 80 nm thickness. All the devices were tested in the area of the aluminium cathode as well as but also in the area of the **POF** polymer only, using gallium-indium eutectic as electrode.

Table S2. Conditions used in building OLEDs in phase 2

Sample	PEDOT:PSS<br>Speed/Acceleration /Annealing temperature	POF<br>Speed/Acceleration /Annealing temperature	Current-luminance-voltage curve
**21**	2000/4000/80	2000/4000/50	Fig.S2a
**22**	2000/4000/80	3000/4000/50	Fig.S2b
**23**	2000/4000/80	4000/4000/50	
**24**	3000/4000/80	2000/4000/80	Fig.S2c
**25**	3000/4000/80	3000/4000/80	

**26**	3000/4000/80	4000/4000/80	
**27**	NA	1500/4000/80	Fig.S2d
**28**	NA	2000/4000/80	Fig.S2e
**29**	NA	2500/4000/80	
**30**	NA	3000/4000/80	
**31**	NA	3500/4000/80	
**32**	NA	4000/4000/80	

NA: not applicable

The devices obtained in Phase 1 and Phase 2 were tested in a dark room. Platinum and chromium electrodes were deposited by cathodic pulverization with a Q150T Cvorum Technologies Sputter Coater.

OLED testing

In order to select the optimum conditions for using **POF** as an active substrate in OLED building, 32 OLED devices were prepared by varying the solvent used for dissolving the polymer (chloroform or toluene), the speed and the acceleration in the spin coating of the organic layer, the temperature applied in the thermal treatment process, the cathode nature (platinum, chromium, aluminium, gallium-indium amalgam) (Table S1). Only the samples 1-6 and 18,19 showed typical OLED behaviour (Figure S2). The analysis of the current-luminance-voltage curves revealed that (1) chromium and platinum cathodes are poor candidate for the **POF**, the most probably because of the high work function difference between them and ITO; (2) aluminium appeared to be a promising cathode as it assured the OLED function from a turn-on voltage of 7 V, however the devices suffered a short circuit at further voltage increasing, most likely do to the presence of impurities; (3) the direct contact through gallium-indium eutectic showed functionality for a threshold voltage of 8 V; (4) microscopic inspection showed that the use of solvents with higher boiling point led to organic layers of better quality; (5) the use of PEDOT:PSS didn’t substantially improved the luminance.

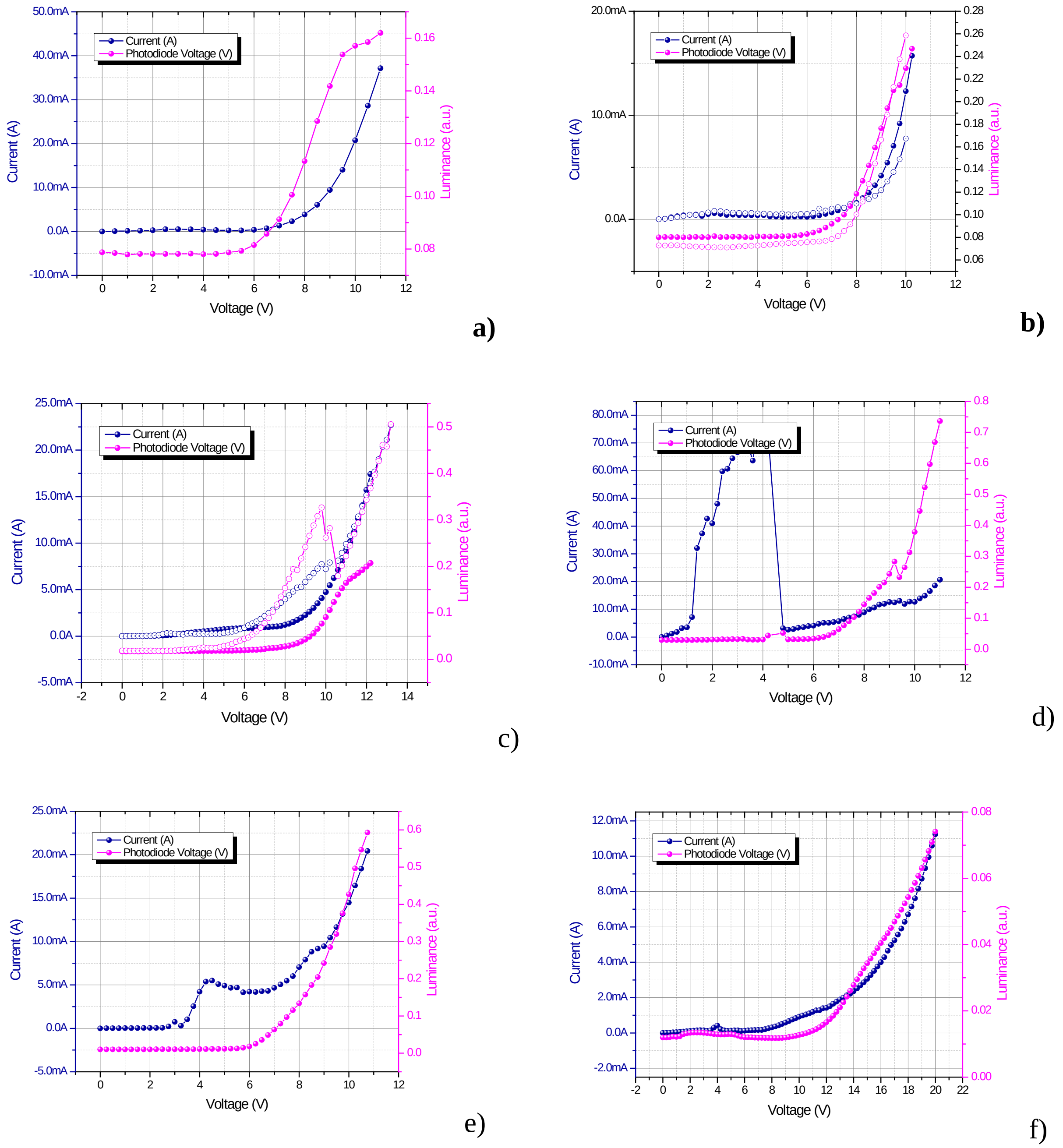

Figure S2. Current-luminance-voltage curves for the **1 – 6** samples

The phase 1 investigation of the OLED devices revealed the aluminium as the most promising superior electrode, and solvents with higher boiling point as the best choice for obtaining organic films of good quality. In the light of these results, a new series of OLEDs were designed using aluminium as cathode and casting the active substrate of **POF** from

chlorobenzene solution (1%). To determine the influence of the active substrate thickness on the OLED performance, various spinning rates were used during the spin coating process (Table S2). The testing investigation on a series of 12 devices (21-31) showed short circuit for 11 samples for which aluminium was the electrode, while the 12-th showed OLED behaviour with a threshold voltage at 10 V but short circuit at 12 V. On the other hand, all the devices showed OLED behaviour when gallium-indium eutectic was used as electrode, but those for which the **POF** was casted at a spinning rate higher than 2000 rpm showed short circuit after a short operation time (Figure S3). This was in line with the better quality of the thicker organic films obtained at lower spinning rate, as the microscopic images demonstrated. Interestingly enough, the best results were obtained for the OLED devices prepared without a PEDOT:PSS substrate and thicker films of **POF** (OLEDs 27 and 28), when electroluminescence intensity of almost one order magnitude higher was observed. It was concluded that the sputter-coating was not a proper process for aluminium deposition and thicker **POF** films are desirable for better performances.

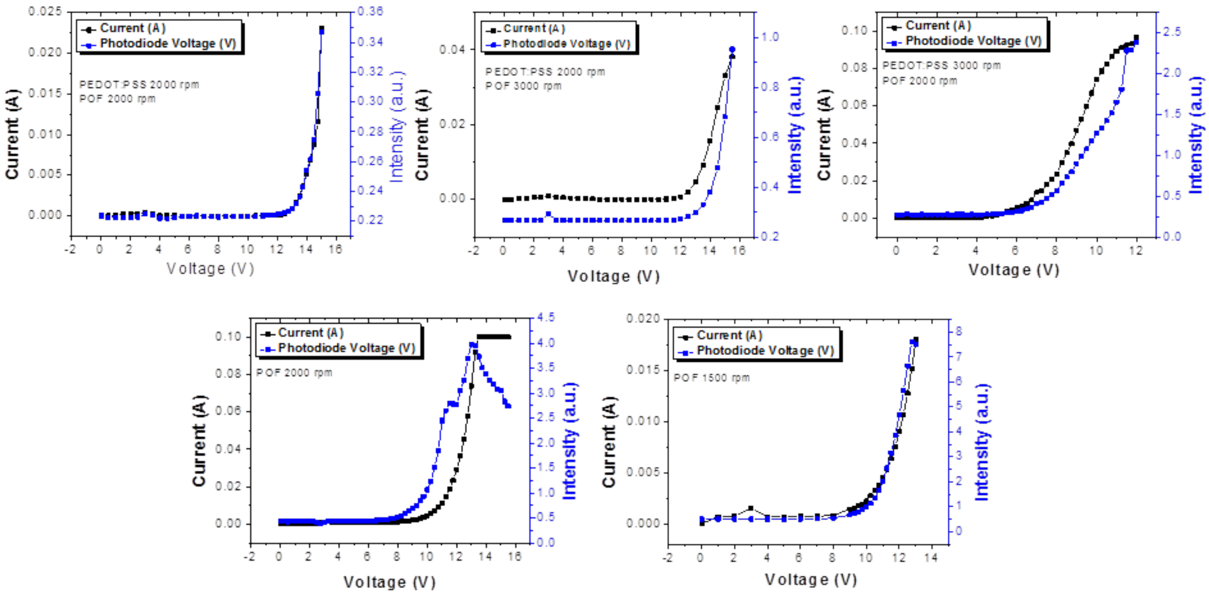

Figure S3. Current-luminance-voltage curves of the OLED devices: **21**, **22**, **24**, **27**, **28** (from left to right)

With these conclusions in mind, the next step was building OLED devices into a glove box, depositing the **POF** polymer from chlorobenzene at a low spinning rate of 2000 rpm and depositing aluminium by thermal evaporation. 9 such devices were built; varying the thickness of

the organic films and of the aluminium cathode. All of the OLED devices were functional. The current-luminance-voltage curves of the OLEDs were given in Figure S4.

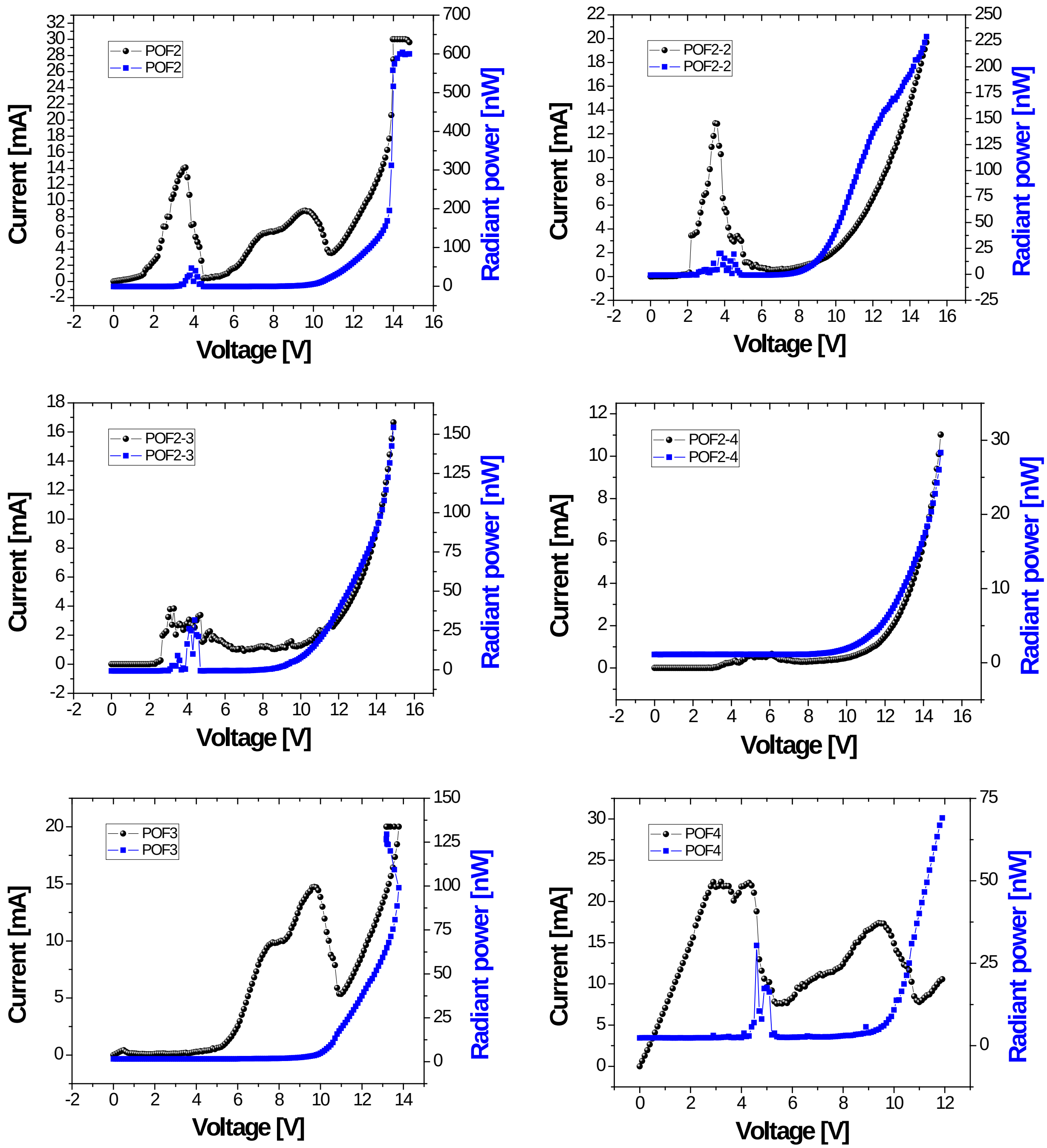

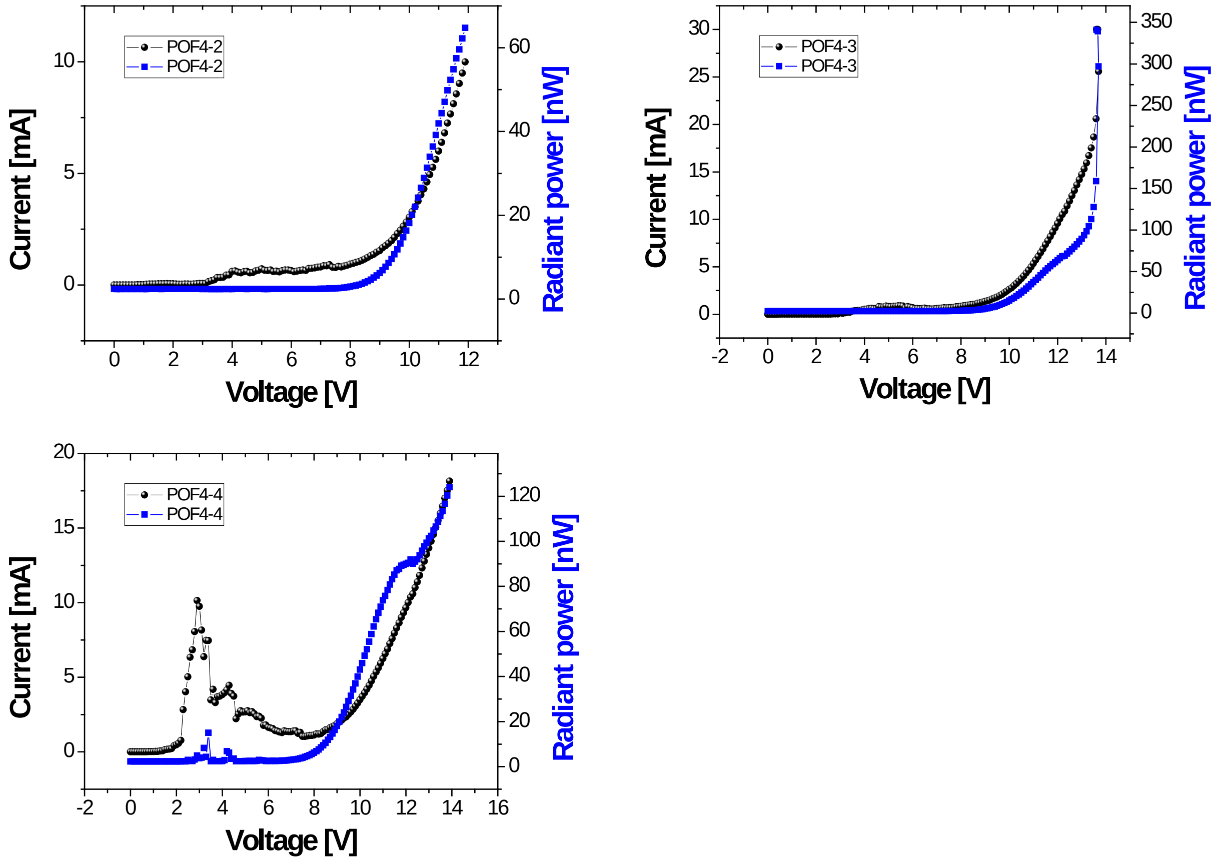

Figure S4. Current-luminance-voltage curves for OLEDs built in clean room and the image of an OLED device
